# *Notes from the Field:* Measles Outbreak — Central Ohio, 2022–2023

**DOI:** 10.15585/mmwr.mm7231a3

**Published:** 2023-08-04

**Authors:** Elizabeth C. Tiller, Nina B. Masters, Kelley L. Raines, Adria D. Mathis, Stephen N. Crooke, Rebecca C. Zwickl, Gavin K. French, Emily R. Alexy, Elizabeth M. Koch, Naomi E. Tucker, Elizabeth M. Wilson, Tiffany S. Krauss, Erica Leasure, Jeremy Budd, Laurie M. Billing, Courtney Dewart, Kara Tarter, Kristen Dickerson, Radhika Iyer, Alexandria N. Jones, Katia C. Halabi, Matthew C. Washam, David E. Sugerman, Mysheika W. Roberts

**Affiliations:** ^1^Epidemic Intelligence Service, CDC; ^2^Columbus Public Health, Columbus, Ohio; ^3^Division of Viral Diseases, National Center for Immunization and Respiratory Diseases, CDC; ^4^Ohio Department of Health; ^5^Division of State and Local Readiness, Office of Readiness and Response, CDC; ^6^Franklin County Public Health, Columbus, Ohio; ^7^Nationwide Children’s Hospital, Columbus, Ohio.

On November 5, 2022, Columbus Public Health, Ohio and the Ohio Department of Health were notified of two children aged 2 years who were admitted to a central Ohio hospital with rash, fever, cough, and congestion, suggestive of measles. Both children were undergoing medical evaluation and treatment for other etiologies before measles was considered in the differential diagnosis. Neither child had received measles, mumps, and rubella (MMR) vaccine, and neither had known contact with a person with measles. Each patient subsequently received a positive measles real-time reverse transcription–polymerase chain reaction (RT-PCR) test result. Neither child had traveled internationally, but during June 12–October 8, 2022, four internationally imported measles cases had been confirmed among unvaccinated Franklin County, Ohio residents who had traveled to areas in East Africa where measles outbreaks were ongoing (*1*). Investigation of the U.S.-acquired measles cases identified additional measles cases, and local and state health departments confirmed a community outbreak on November 9, 2022. During this community measles outbreak in central Ohio, 85 locally acquired measles cases were confirmed with rash onsets during October 22–December 24, 2022; however, no definitive link to the previous international importations was established. The outbreak was declared over on February 4, 2023, 42 days (two measles incubation periods) after the last reported case.

## Investigation and Outcomes

Suspected measles cases were investigated through patient, health care provider, and child care facility interviews, medical records review, and consultation with health care providers. Nasopharyngeal swab and serum specimens were collected in accordance with recommendations.* The 85 confirmed cases included 78 in Franklin County, two in Madison County, and one each in Clark, Fairfield, Richland, Ross, and Union counties, all counties within central Ohio. The Ohio Department of Health Public Health Laboratory performed RT-PCR testing of specimens from 193 persons during the outbreak; 74 (87%) measles cases were laboratory-confirmed,^ †^ and the remaining 11 (13%) were epidemiologically linked to confirmed cases. Among 65 genotyped specimens, all were genotype B3. The median patient age was 1 year (range = 6 months–15 years). Eighty (94%) patients had not received MMR vaccine. Sixty (71%) patients were aged ≥1 year and age-eligible for routine MMR vaccination,^§^ but only three (5%) had documentation of receipt of 1 MMR vaccine dose at the time of infection^¶^; vaccination status of one (2%) patient was unknown.

Forty-four (52%) of the 85 measles patients experienced complications, including otitis media (33; 39%), diarrhea (22; 26%), and pneumonia (seven; 8%); 36 (42%) patients were hospitalized, predominately for dehydration.** The median length of hospitalization was 3 days (range = 1–7 days). Twelve hospitalized patients had coinfections with other respiratory pathogens (e.g., **respiratory syncytial virus **[**RSV**]).^††^ No deaths were reported.

Reported exposure locations for measles included five health care facilities accounting for 32 (38%) of 85 total cases, four child care facilities with 22 (26%) cases, and households with 17 (20%) cases (Figure). This outbreak occurred during a peak in emergency department visits for COVID-19, RSV, and influenza (*2*).

**FIGURE:**
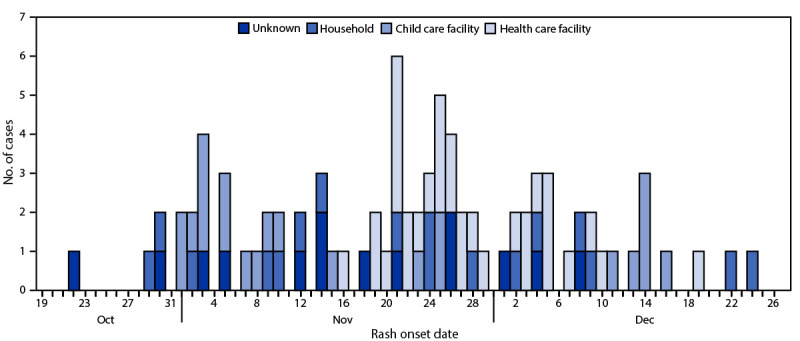
Measles cases, by rash onset date and exposure locations (N = 85) — Central Ohio, October–December 2022

Columbus Public Health and Franklin County Public Health departments identified 739 local contacts who were unvaccinated or had unknown vaccination status and required quarantine; 446 (60%) were successfully enrolled in active monitoring. Among these 446 persons, 43 (10%) developed measles during quarantine, 28 (65%) of whom were reached before rash onset. Postexposure prophylaxis was administered to 59 contacts, including eight (14%) who received MMR vaccine, and 51 (88%) who received immune globulin.^§§^ Two children who received MMR vaccine and none who received immune globulin were later diagnosed with measles.

## Preliminary Conclusions and Actions

Ohio previously experienced a measles outbreak in 2014. During 2021–2022, kindergarten-entry 2-dose MMR vaccination coverage in Ohio (88.3%) was approximately 5% lower than the national estimate of 93.0% (*3*). Although measles was declared eliminated in the United States in 2000,^¶¶^ it remains endemic in many countries, and internationally imported cases continue to be associated with outbreaks among undervaccinated, close-knit communities in the United States (*4*). This outbreak was characterized by young median patient age, low rates of MMR vaccination, and high rates of respiratory coinfection, with twice the hospitalization rate reported among previous measles cases in the United States (*5*).

This outbreak serves as a reminder that health care facilities, medical providers, and child care facilities serving undervaccinated populations should maintain vigilance for measles and emphasize the importance of timely MMR vaccination. Sustaining elimination of measles in the United States will require continued high 2-dose MMR vaccination coverage in all communities.

## Footnotes

* https://www.cdc.gov/vaccines/pubs/surv-manual/chpt07-measles.html

† The 74 laboratory-confirmed cases included 71 with RT-PCR–positive results and three with immunoglobulin M–positive results.

§ The Advisory Committee on Immunization Practices recommends 2 routine MMR vaccine doses for children, with the first dose administered at age 12–15 months and the second dose at age 4–6 years, before school entry.

¶ One patient received MMR vaccine 35 months before known exposure; one patient received MMR vaccine ≤72 hours after known exposure; and one patient with an unknown exposure date received MMR vaccine 9 days before rash onset.

** Reasons for admission obtained from medical records.

†† Identified respiratory pathogens included adenovirus, group A Streptococcus, influenza A, rhinovirus, enterovirus, RSV, and SARS-CoV-2.

§§ MMR vaccine administered ≤72 hours after exposure; immune globulin administered ≤6 days after exposure.

¶¶ The World Health Organization defines measles elimination as the absence of endemic measles virus transmission in a defined geographic area (e.g., region or country) for ≥12 months in the presence of a surveillance system that has been verified to be performing well. https://www.cdc.gov/measles/elimination.html

## References

[1] World Health Organization. Immunization analysis and insights. Provisional monthly measles and rubella data. Geneva, Switzerland: World Health Organization; 2023. Accessed March 13, 2023. https://www.who.int/teams/immunization-vaccines-and-biologicals/immunization-analysis-and-insights/surveillance/monitoring/provisional-monthly-measles-and-rubella-data

[2] CDC. NCIRD surveillance. National emergency department visits for COVID-19, influenza, and respiratory syncytial virus. Atlanta, GA: US Department of Health and Human Services, CDC; 2023. Accessed March 13, 2023. https://www.cdc.gov/ncird/surveillance/respiratory-illnesses/index.html

[3] Seither R, Calhoun K, Yusuf OB, Vaccination coverage with selected vaccines and exemption rates among children in kindergarten—United States, 2021–22 school year. MMWR Morb Mortal Wkly Rep 2023;72:26–32. 10.15585/mmwr.mm7202a2 36634005 PMC9869733

[4] Mathis AD, Clemmons NS, Redd SB, Maintenance of measles elimination status in the United States for 20 years despite increasing challenges. Clin Infect Dis 2022;75:416–24. 10.1093/cid/ciab979 34849648 PMC11288340

[5] Gastañaduy PA, Funk S, Lopman BA, Factors associated with measles transmission in the United States during the postelimination era. JAMA Pediatr 2020;174:56–62. 10.1001/jamapediatrics.2019.4357 31738391 PMC6865326

